# Editorial for the Special Issue: Oral Immunology and Periodontitis

**DOI:** 10.3390/pathogens11050564

**Published:** 2022-05-10

**Authors:** Ulvi K. Gürsoy

**Affiliations:** Department of Periodontology, Institute of Dentistry, University of Turku, Lemminkäisenkatu, 2, 20520 Turku, Finland; ulvgur@utu.fi

The two most common forms of oral infectious diseases are caries and periodontal diseases. Periodontal diseases, namely gingivitis and periodontitis, occur as inflammatory responses against the constant challenges of infections. Cellular components of the oral immune response are very similar to systemic immune responses; however, the interactions between oral bacteria, oral hard tissues, resident cells of the periodontium, immune cell activation, and genetic determinants make oral immune responses unique [1].

This Special Issue, entitled “Oral Immunology and Periodontitis”, is composed of seven original articles and four reviews and aims to discuss different aspects of oral immune responses in the pathogenesis of periodontitis. In their original article, Kasnak et al. [2] raised the question of whether matrix metalloproteinase (MMP)-3-1171 5A/6A polymorphism has an association with gingival crevicular fluid (GCF) MMP-3 levels and periodontal destruction. As a result, they stated that an association between GCF MMP-3 levels and periodontitis occurred but not with the single-nucleotide polymorphism. According to Esberg et al. [3] there are global antibody patterns for oral bacteria or potential population time trends. They observed an increase in IgG towards *Streptococcus salivarius* and *Streptococcus sanguinis* in the younger population and towards *Aggregatibacter actinomycetemcomitans*, *Filifactor alocis*, and *Streptococcus mutans* in the elderly population. Gursoy et al. [4] tested the ability of human milk oligosaccharides to activate human beta-defensin (hBD) expressions and found that 2′-fucosyllactose and 3-fucosyllactose have the ability to stimulate hBD-2 expression. Lundtorp-Olsen et al. [5] tested their hypotheses that the consumption of probiotics counteracts the negative effects of sugar stress on oral homeostasis. They demonstrated that the combined consumption of xylitol and probiotics has an impact on salivary microbiota composition. In their second study, Lundtorp-Olsen et al. [6] evaluated the compositional stability of supragingival microbiota against the regular use of probiotics and found no augmenting effect of probiotics on the supragingival microbiota. Elmanfi et al. [7] analyzed the regulatory roles of bacterial signaling molecules and cyclic dinucleotides on human gingival fibroblast behavior. They showed that bacterial cyclic dinucleotides interacted with lipopolysaccharide-mediated cellular responses. Finally, in their 12-week follow-up study, Gursoy et al. [8] found that the total salivary protease activity could be used as a biomarker of unresponsive tissue responses against periodontal treatment.

In their reviews, Celik and Kantarci [9] emphasized vascular changes and hypoxia while discussing systemic disease–periodontitis interactions. Grant [10] discussed the role of pyruvate kinase in the pathogenesis of periodontitis. Elmanfi et al. [11] explored the possible role of bacterial cyclic dinucleotides in the pathogenesis of periodontitis. Finally, Mei et al. [12] explained the systemic effects of the well-known periodontal pathogen, *Porphyromonas gingivalis*.

Periodontitis is a multifactorial disease with strong bacterial and host components. Understanding the role of immune regulation during the initiation, progression, and remission of periodontitis will help researchers and clinicians to develop new and novel diagnostic and therapeutic techniques.
